# Hybrid Mamba–Graph Fusion with Multi-Stage Pseudo-Label Refinement for Semi-Supervised Hyperspectral–LiDAR Classification

**DOI:** 10.3390/s26031005

**Published:** 2026-02-03

**Authors:** Khanzada Muzammil Hussain, Keyun Zhao, Sachal Perviaz, Ying Li

**Affiliations:** School of Computer Science, Northwestern Polytechnical University, Xi’an 710129, China; muz1@mail.nwpu.edu.cn (K.M.H.); kyzhao@mail.nwpu.edu.cn (K.Z.); sachalpervaiz@mail.nwpu.edu.cn (S.P.)

**Keywords:** data fusion, semi-supervised learning, pseudolabel refinement, Mamba state-space model, graph neural network, multimodal classification

## Abstract

**Highlights:**

**What are the main findings?**

- Novel Hybrid Architecture for HSI–LiDAR Fusion: We propose HMGF-Net, a unified multimodal network combining a spectral–spatial CNN (HSI) and a multi-scale CNN (LiDAR) with a Mamba state-space sequence module and a graph-based fusion layer to capture both local and long-range contextual features across modalities.
- Multi-Stage Pseudo-Label Refinement: We develop a three-stage semi-supervised training pipeline that progressively refines pseudolabels using confidence-based filtering, spatial–spectral smoothing via a KNN graph, and graph-consistency checks, effectively denoising labels and stabilizing training with very limited ground truth.

**What is the implication of the main findings?**

- State-of-the-Art Performance with Limited Labels:The proposed approach achieves superior classification accuracy on three benchmark hyperspectral–LiDAR datasets (Houston2013, Augsburg, Trento), outperforming eight recent state-of-the-art methods. Notably, HMGF-Net attains higher overall accuracy (up to 92–99%) and average accuracy than competitors, with pronounced gains in low-label regimes (only ten labeled samples per class).
- Synergy of Sequence Modelling and Graph Reasoning: Our results demonstrate that integrating long-range sequence modeling (Mamba) with graph-based context propagation yields smoother classification maps and better class discrimination. The hybrid approach reduces salt-and-pepper noise and improves boundary delineation in the predicted maps, highlighting its effectiveness for detailed sensor data analysis.

**Abstract:**

Semi-supervised joint classification of Hyperspectral Images (HSIs) and LiDAR-derived Digital Surface Models (DSMs) remains challenging due to scarcity of labeled pixels, strong intra-class variability, and the heterogeneous nature of spectral and elevation features. In this work, we propose a Hybrid Mamba–Graph Fusion Network (HMGF-Net) with Multi-Stage Pseudo-Label Refinement (MS-PLR) for semi-supervised hyperspectral–LiDAR classification. The framework employs a spectral–spatial HSI backbone combining 3D–2D convolutions, a compact LiDAR CNN encoder, Mamba-style state-space sequence blocks for long-range spectral and cross-modal dependency modeling, and a graph fusion module that propagates information over a heterogeneous pixel graph. Semi-supervised learning is realized via a three-stage pseudolabeling pipeline that progressively filters, smooths, and re-weights pseudolabels based on prediction confidence, spatial–spectral consistency, and graph neighborhood agreement. We validate HMGF-Net on three benchmark hyperspectral–LiDAR datasets. Compared with a set of eight state-of-the-art (SOTA) baselines, including 3D-CNNs, SSRN, HybridSN, transformer-based models such as SpectralFormer, multimodal CNN–GCN fusion networks, and recent semi-supervised methods, the proposed approach delivers consistent gains in overall accuracy, average accuracy, and Cohen’s kappa, especially in low-label regimes (10% labeled pixels). The results highlight that the synergy between sequence modeling and graph reasoning in combination with carefully designed pseudolabel refinement is essential to maximizing the benefit of abundant unlabeled samples in multimodal remote sensing scenarios.

## 1. Introduction

Hyperspectral Images (HSI) provide dense spectral information across hundreds of narrow bands, and have become indispensable for material identification and land cover analysis. However, issues such as spectral redundancy, nonlinear mixing, and high dimensionality continue to challenge robust spectral–spatial modeling, motivating the development of advanced learning strategies for high-dimensional remote sensing data [1,2]. Recent deep learning approaches have improved HSI classification through joint spatial–spectral feature extraction, hybrid CNN–attention architectures, and lightweight networks designed for small-sample settings [3,4,5].

To address the limitations of spectral information alone, complementary modalities such as Light Detection and Ranging (LiDAR) have been extensively integrated with HSI. LiDAR provides elevation and structural cues that enhance edge delineation and mitigate spectral ambiguity. Numerous studies have demonstrated that combining HSI and LiDAR significantly improves classification accuracy, particularly in heterogeneous or urban environments [6,7,8]. Recent advances include dual-branch transformers, hypergraph networks, and cross-attention fusion modules that model heterogeneous spectral–elevation interactions more effectively [9,10,11]. In addition to dual-modality setups, multisensor fusion frameworks now exploit combinations of hyperspectral, multispectral, radar, and LiDAR data to achieve more stable and generalizable scene understanding [12,13].

Despite this progress, multimodal HSI–LiDAR fusion remains difficult under limited supervision. HSI suffers from large intra-class spectral variance and sensitivity to noise, while LiDAR exhibits irregular sampling patterns and modality-specific distortions. Effective fusion requires bridging disparate feature spaces while preserving modality-specific advantages. Deep HSI classifiers based on random-patch learning, dense residual transfer, and spectral–spatial CNNs offer improved robustness [3,4,14], yet most multimodal fusion networks assume abundant labeled samples and degrade sharply when labels are scarce [15,16].

To address this, Semi-Supervised Learning (SSL) has gained importance in HSI classification. Modern SSL methods integrate unlabeled samples through consistency constraints, pseudolabel refinement, adversarial learning, and hybrid generative–discriminative models [17,18,19]. In addition, multiscale refinement strategies and cost-aware learning mechanisms have been shown to substantially improve label efficiency in low-annotation scenarios [15,20]. Nonetheless, applying SSL to multimodal fusion remains challenging, as pseudolabel noise can propagate between modalities, causing semantic drift unless cross-modal agreement and structural consistency are jointly enforced [21].

Meanwhile, long-range modeling advances have reshaped deep learning for remote sensing. Transformer-based architectures and graph neural networks have demonstrated strong spectral–spatial reasoning capabilities, but remain limited by computational complexity when applied to high-resolution hyperspectral cubes [2,16]. Recently, visual State-Space Models (SSMs), particularly Mamba-inspired architectures, have emerged as efficient alternatives that capture long-range dependencies with linear complexity. RS3Mamba and ConvMambaSR show excellent performance in segmentation and super-resolution tasks, highlighting the potential of SSMs for hyperspectral sequence modeling [22,23]. Additional work in SSM-driven remote sensing indicates improved efficiency, stability, and scalability compared to transformer-based models [24,25,26].

Despite these advances, existing state-space and graph-based approaches for remote sensing classification exhibit several limitations. Current Mamba-inspired architectures such as RS3Mamba [22] and ConvMambaSR [23] employ fixed scanning strategies over the spatial grid that do not adapt to irregular object boundaries in heterogeneous scenes, potentially overlooking critical cross-modal relationships at class transitions where spectral and elevation discontinuities do not align. While graph neural networks have shown promise for HSI classification through hypergraph convolutions [8] and cross-attention mechanisms [11], they face well-documented over-smoothing effects when stacked beyond two or three layers, and incur significant computational overhead when constructing dense pixel graphs over high-resolution hyperspectral cubes [2,16]. Most critically, neither paradigm alone addresses the challenge of pseudolabel noise propagation in semi-supervised multimodal learning. Erroneous predictions generated from one modality can corrupt feature representations through the fusion mechanism, leading to semantic drift during iterative self-training [21]. These limitations motivate the design of HMGF-Net, which combines efficient state-space sequence modeling with graph-based consistency verification specifically targeted at pseudolabel quality control. Motivated by these developments, we propose HMGF-Net. a Hybrid Mamba–Graph Fusion network for semi-supervised HSI–LiDAR classification. The proposed network integrates (i) spectral–spatial HSI encoding, (ii) multiscale LiDAR structural modeling, (iii) selective state-space sequence modeling for efficient long-range dependency capture, and (iv) graph-guided multimodal fusion. To mitigate pseudolabel noise in SSL, we introduce Multi-Stage Pseudo-Label Refinement (MS-PLR), a mechanism that applies confidence filtering, spatial–spectral smoothing, and graph-consistency propagation. Together, these components enable HMGF-Net to achieve robust and stable performance even under extremely limited labeled data.

These observations collectively motivate the design of a unified architecture capable of addressing the interconnected challenges of multimodal learning under limited labels. Existing methods rarely combine spectral–spatial HSI encoding, LiDAR structural modeling, efficient long-range sequence learning, graph reasoning, and systematic pseudolabel refinement into a single coherent framework. Moreover, multimodal SSL remains vulnerable to inconsistent pseudolabel predictions, which can propagate uncertainty and destabilize training. Additionally, high-dimensional hyperspectral sequences require an efficient long-range modeling approach that avoids the computational burden of transformers. These challenges form the foundation for our proposed methodology.

To address these intertwined challenges, we introduce HMGF-Net, a Hybrid Mamba–Graph Fusion Network equipped with an end-to-end Multi-Stage Pseudo-Label Refinement (MS-PLR) mechanism. In contrast to existing multimodal approaches, HMGF-Net integrates spectral–spatial representation learning for hyperspectral data, multiscale geometric modeling for LiDAR elevation cues, and efficient long-range dependency modeling through the Mamba selective state-space paradigm. These encoded features are subsequently processed within a graph-based fusion network that captures cross-modal relational structure and enhances contextual reasoning. Finally, the MS-PLR pipeline progressively refines pseudolabels through confidence filtering, spatial–spectral smoothing, and graph-consistency propagation, enabling the network to suppress noise, reinforce cross-modal stability, and achieve high classification accuracy even in demanding low-label conditions.

The main contributions of this work are summarized below:

1. We present HMGF-Net, a unified multimodal architecture that integrates a 3D–2D spectral–spatial CNN encoder for hyperspectral data, a multiscale CNN for LiDAR elevation modeling, and Mamba-based selective state-space modeling for efficient long-range dependency learning. This design combines local spectral–spatial feature extraction with global sequence modeling, enabling a more expressive and computationally efficient multimodal representation than conventional CNN- or transformer-based approaches.
2. We introduce a graph-guided multimodal fusion mechanism that aligns hyperspectral and LiDAR features using relational modeling based on spectral similarity, spatial proximity, and elevation-informed neighborhood structure. This graph-based fusion strategy promotes more coherent cross-modal interactions by preserving geometric continuity and spectral–spatial relationships, thereby enabling the network to integrate complementary modality information more effectively than concatenation, attention-only fusion, or shallow multimodal alignment methods.
3. We develop a Multi-Stage Pseudo-Label Refinement (MS-PLR) framework designed to stabilize semi-supervised learning through progressive noise suppression. The refinement process incorporates confidence filtering, spatial–spectral neighborhood smoothing, and graph consistency propagation to reduce the influence of unreliable predictions. This enables more reliable pseudolabel supervision in low-label scenarios, preventing semantic drift and improving training stability by ensuring that refined labels remain structurally consistent with both spectral–spatial patterns and elevation cues.

## 2. Materials and Methods

### Datasets

To assess the effectiveness of the proposed approach, three publicly accessible multisensor remote sensing image classification datasets are utilized as experimental datasets: the Houston2013, Trento, and Augsburg datasets. Comprehensive parameters are shown in Table 1.

Houston2013 dataset. The Houston2013 dataset was captured using the ITRES CASI-1500 (ITRES Research Limited, Calgary, AB, Canada) sensor over the University of Houston campus and its surrounding urban area in Houston, Texas, USA in 2012. This dataset includes both HSI and LiDAR DSM data. The spatial dimensions of the dataset are 349 × 1905, with a spatial resolution of approximately 2.5 m. The HSI data consist of 144 spectral bands, covering the wavelength range from 380 to 1050 nm. The LiDAR data provide elevation information for ground features. The land cover is categorized into fifteen types: Healthy Grass, Stressed Grass, Synthetic Grass, Trees, Soil, Water, Residential, Commercial, Road, Highway, Railway, Parking Lot 1, Parking Lot 2, Tennis Court, and Running Track.

Augsburg dataset. The Augsburg dataset consists of paired HSI and LiDAR DSM data; the HSI data were collected using the HySpex (Norsk Elektro Optikk AS, Skedsmokorset, Norway) sensor, while the LiDAR DSM data were obtained with the DLR-3K sensor (German Aerospace Center, Oberpfaffenhofen, Germany). This dataset was acquired over Augsburg, Germany, which is an urban environment. The spatial dimensions of the Augsburg dataset are 332 × 485, with a spatial resolution of approximately 30 m. The HSI data includes 180 spectral bands, spanning the wavelength range of 0.4 to 2.5 µm. The LiDAR DSM data provides 3D elevation information for surface features. The dataset comprises seven land cover categories with varying sample distributions.

Trento Dataset. The Trento dataset is an HSI–LiDAR pair dataset; the HSI data were collected by an AISA Eagle (Specim, Spectral Imaging Ltd., Oulu, Finland) sensor, while the LiDAR digital surface model (DSM) data were acquired by an Optech ALTM 3100EA (Teledyne Optech, Vaughan, ON, Canada) sensor. The dataset was captured over a rural area south of the city of Trento, Italy. The Trento dataset has a spatial dimension of 166 × 600 with a spatial resolution of approximately 1 m. The HSI data in the Trento dataset consist of 63 spectral bands, with wavelengths ranging from 420 to 990 nm. The LiDAR DSM data provide elevation information of ground features. The land cover is classified into six categories: Apple Trees, Buildings, Ground, Woods, Vineyard, and Roads.

## 3. Methods

**Figure 1:**
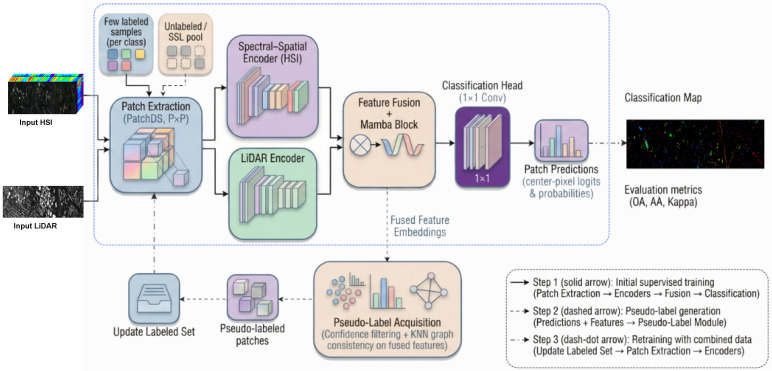
Overall framework of the proposed HMGF-Net with MS-PLR training strategy.

To address the challenge of limited labeled samples in multi-source remote sensing image classification, we propose a Heterogeneous Multimodal Graph Fusion Network (HMGF-Net) trained with a Multi-Stage Progressive Learning Refinement (MS-PLR) strategy. The proposed framework consists of two complementary components: (1) the HMGF-Net architecture, which effectively integrates hyperspectral and LiDAR features through modality-specific encoders and a Mamba-based fusion module, and (2) the MS-PLR training strategy that leverages unlabeled data through graph-regularized pseudolabeling. The overall framework is illustrated in Figure 1, and the key notations used throughout this paper are summarized in Table 2.

### 3.1. Framework Overview

As shown in Figure 1, the proposed framework integrates the HMGF-Net architecture with the MS-PLR training strategy. The HMGF-Net architecture comprises three main components: (1) a dual-branch feature extraction module with modality-specific encoders, (2) a Mamba-based feature fusion module for cross-modal interaction, and (3) a classification head for land cover prediction. The MS-PLR training strategy employs a three-stage paradigm to maximize the utilization of both labeled and unlabeled samples:

Stage 1 (Supervised Pretraining): HMGF-Net is initially trained on the small labeled set $D_{l}$ using cross-entropy loss. The forward path consists of patch extraction, modality-specific encoders, Mamba-based feature fusion, and classification head. This stage establishes the initial feature representations.

Stage 2 (Pseudolabel Generation): Using the pretrained HMGF-Net, we generate predictions for all unlabeled samples in $D_{u}$. High-confidence predictions are filtered through dynamic thresholding and validated via KNN graph consistency checking. Only samples that pass both criteria are accepted as pseudolabels.

Stage 3 (Semi-Supervised Refinement): HMGF-Net is fine-tuned on the augmented dataset $D_{aug}=D_{l}\cup P$, where P denotes the validated pseudolabel set. A reduced learning rate prevents catastrophic forgetting of the knowledge learned in Stage 1.

### 3.2. HMGF-Net Architecture

The HMGF-Net architecture is designed for effective multimodal feature extraction and fusion. We describe each architectural component in detail below.

#### 3.2.1. Dual-Branch Feature Extraction Module

**Figure 2:**
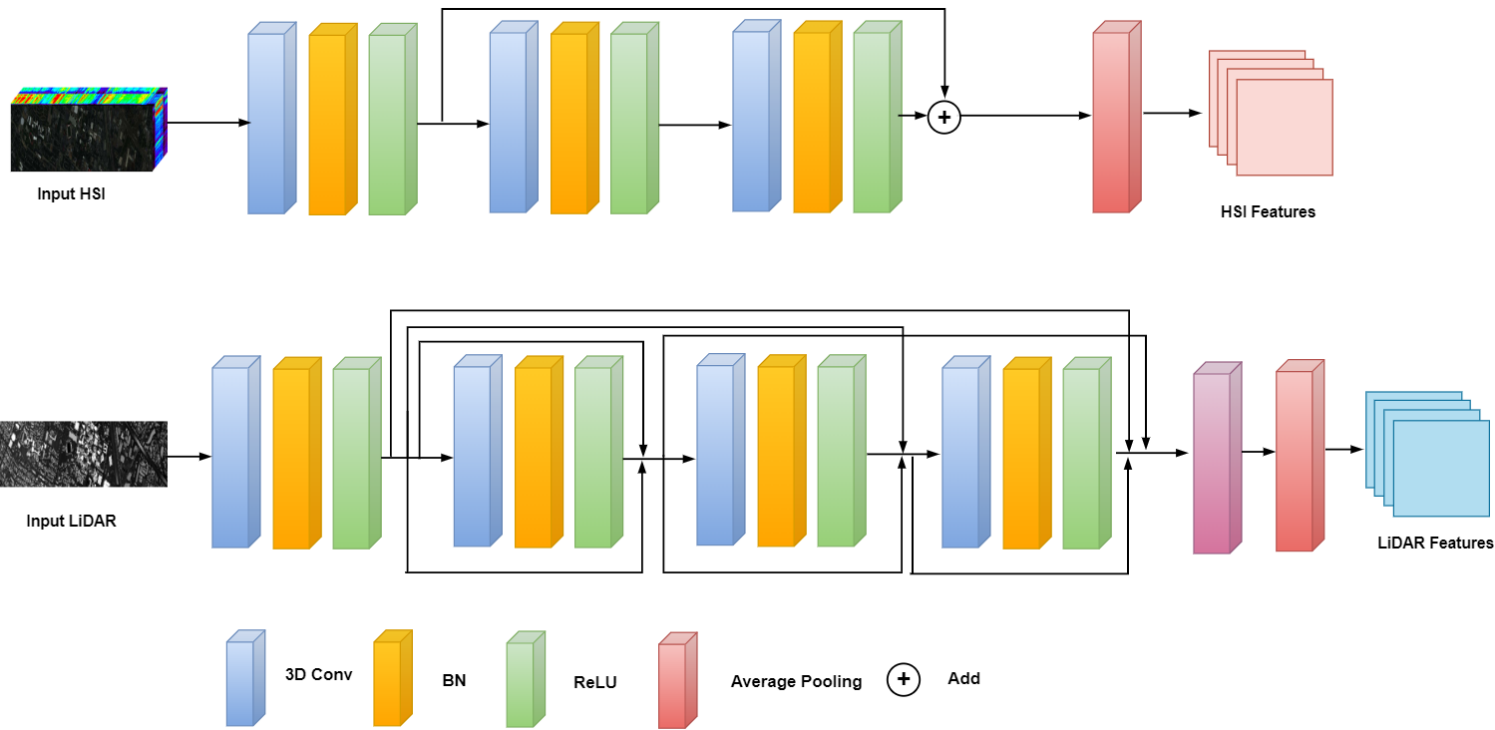
Dual-branch feature extraction module.

For multi-source remote sensing data, we develop a dual-branch architecture with customized connectivity mechanisms specifically designed for the distinct characteristics of HSI and LiDAR data. Figure 2 illustrates the feature extraction module.

The HSI encoder employs 3D convolutions to jointly capture spectral correlations and spatial context. For an input patch $P_{H}\in R^{1\times C\times P\times P}$, we apply three cascaded 3D convolutional layers with spectral kernel sizes of 7, 5, and 3, progressively reducing spectral dimensionality while extracting hierarchical features. Each layer incorporates batch normalization and ReLU activation to stabilize training and introduce non-linearity.

Considering the spectral–spatial complexity and potential noise in spatial dimensions, we enhance the standard 3D CNN with residual learning. The residual block incorporates skip connections that allow direct propagation of spectral features across layers, which helps preserve critical spectral information while alleviating gradient vanishing issues. It can be expressed as

(1) $$F_{H}^{\left(l+2\right)}=H_{H}\left(F_{H}^{\left(l\right)}\right)+F_{H}^{\left(l\right)},$$

where $F_{H}^{\left(l\right)}$ represents the input of the *l*-th layer and $H_{H}\left(\cdot \right)$ represents the mapping function. The 3D features are reduced via spectral average pooling and a 2D convolution to produce $Z_{H}\in R^{D\times P\times P}$, where D=64.

Because LiDAR data adopt the Digital Surface Model format, which has local correlations and sparsity, we use a dense connection method in which features of each layer are connected to all previous layers. This effectively leverages local correlations and enhances the re-usability and representational power of features:

(2) $$F_{L}^{\left(l+2\right)}=H_{L}^{\left(l+2\right)}\left(\left[F_{L}^{\left(0\right)},F_{L}^{\left(1\right)},\ldots ,F_{L}^{\left(l+1\right)}\right]\right)$$

where [·] denotes concatenation of features from all preceding layers. The LiDAR encoder processes elevation information through three 2D convolutional layers with channel dimensions 1→32→64→64, producing $Z_{L}\in R^{D\times P\times P}$.

#### 3.2.2. Mamba-Based Feature Fusion Module

**Figure 3:**
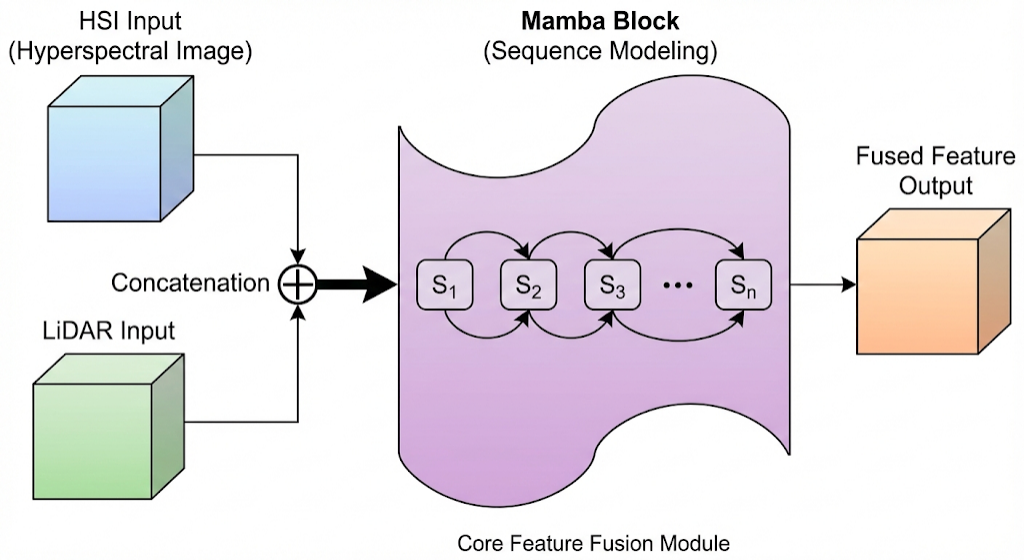
Feature fusion and Mamba sequence modeling module. HSI and LiDAR features are concatenated, reshaped to sequence, processed through GRU gates, and reshaped back to spatial domain. Ellipsis dots (…) indicate omitted intermediate layers for visual clarity.

To capture long-range spatial dependencies and enable effective cross-modal interaction, we propose a Mamba-based state-space fusion module. Unlike attention mechanisms, which have $O(L^{2})$ complexity, the state-space model formulation enables linear-time O(L) sequence modeling while capturing long-range dependencies. The detailed structure is illustrated in Figure 3.

The modality-specific features are first concatenated along the channel dimension to form a unified representation

(3) $$Z_{cat}=\left[Z_{H};Z_{L}\right]\in R^{2D\times P\times P},$$

then the spatial feature map is reshaped into a sequence representation to model spatial positions as sequential tokens:

(4) $$S=\mathrm{Reshape}\left(Z_{cat}\right)\in R^{L\times N}$$

where $L=P^{2}$ is the sequence length and N=2D is the feature dimension. This transformation enables the application of sequential modeling to capture spatial relationships.

Our Mamba block is based on structured state-space sequence models (S4/Mamba), which map input sequences to output sequences through a latent state. The discretized state-space recurrence for each spatial position t∈{1,…,L} is

(5) $$h_{t}=\bar{A}h_{t-1}+{\bar{B}}_{t}x_{t},\;y_{t}=C_{t}h_{t},$$

where $h_{t}\in R^{N_{s}}$ is the latent state with dimension $N_{s}$, $\bar{A}$ is the discretized state transition matrix, and ${\bar{B}}_{t}$, $C_{t}$ are input-dependent projection matrices that enable content-aware sequence modeling. This selective mechanism allows the model to adaptively filter and retain relevant cross-modal information based on input content, which is crucial for fusing heterogeneous HSI spectral and LiDAR elevation features.

The complete Mamba block processes the input sequence through a gating mechanism:

(6) $$Z_{fused}=\mathrm{Reshape}\left(\mathrm{SSM}\left(S\right)⊙\sigma_{g}\left(S\right)\right)\in R^{2D\times P\times P}$$

where SSM(·) denotes the selective state-space operation, $\sigma_{g}$ is the SiLU activation function, and ⊙ represents element-wise multiplication to provide a gating mechanism. The gating allows the network to control information flow, suppressing irrelevant features while enhancing discriminative ones.

#### 3.2.3. Classification Head

The fused features are passed through a 1×1 convolution to produce class logits. The center-pixel prediction is extracted and converted to class probabilities via softmax:

(7) $$p_{k}=\frac{\exp({\hat{y}}_{k})}{\sum_{j=1}^{K}\exp({\hat{y}}_{j})},\;k=1,\ldots ,K$$

where *K* is the number of land cover classes. The center-pixel extraction strategy focuses on the most reliable prediction within each patch, reducing boundary effects.

### 3.3. Multi-Stage Progressive Learning Refinement (MS-PLR) Strategy

While the HMGF-Net architecture provides effective multimodal feature fusion, the limited availability of labeled samples in remote sensing applications motivates the development of a semi-supervised training strategy. The MS-PLR strategy leverages unlabeled data through graph-regularized pseudolabeling to progressively refine the model. This training strategy operates on top of the HMGF-Net architecture and consists of three stages, as outlined in Algorithm 1.
**Algorithm 1** HMGF-Net Training with MS-PLR Strategy**Require:** HSI $X_{H}$, LiDAR $X_{L}$, labeled set $D_{l}$, unlabeled set $D_{u}$
**Ensure:** Trained HMGF-Net model $\theta^{*}$
  1:**Stage 1: Supervised Pretraining**  2:Initialize HMGF-Net parameters θ  3:Train HMGF-Net on $D_{l}$ for $E_{sup}$ epochs using $L_{sup}$  4:**Stage 2: Pseudo-Label Generation**  5:Compute predictions and confidences $\left\{\left({\hat{y}}_{j},c_{j}\right)\right\}$ for $D_{u}$ using HMGF-Net  6:Compute dynamic threshold $\tau_{dyn}$  7:Select candidates: $C\leftarrow \{j:c_{j}\geq \tau_{dyn}\}$  8:Build KNN graph on fused features and compute $\left\{{\bar{p}}_{i}\right\}$  9:Filter by agreement: $P\leftarrow \{i\in C:\arg\max{\bar{p}}_{i}={\hat{y}}_{i}\}$10:**Stage 3: Semi-Supervised Refinement**11:Fine-tune HMGF-Net on $D_{l}\cup P$ for $E_{ssl}$ epochs with $\eta_{ssl}$12:**return** $\theta^{*}$


#### 3.3.1. Stage 1: Supervised Pretraining

HMGF-Net is first trained on labeled data using cross-entropy loss with label smoothing to prevent overconfident predictions:

(8) $$L_{sup}=-\frac{1}{|D_{l}|}\sum_{i}\sum_{k=1}^{K}{\tilde{y}}_{ik}\log(p_{k}^{\left(i\right)})$$

where the smoothed label ${\tilde{y}}_{ik}$ applies smoothing parameter ϵ=0.1. This stage establishes robust initial feature representations that form the foundation for subsequent pseudolabel generation.

#### 3.3.2. Stage 2: Graph-Regularized Pseudolabel Acquisition

**Figure 4:**
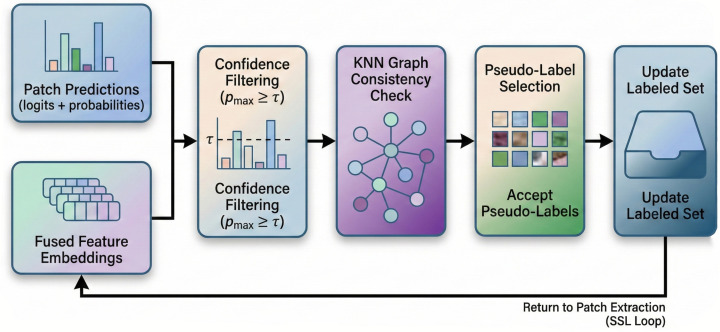
Graph-regularized pseudolabel acquisition pipeline. **Stage 1 (Confidence Filtering):** For each unlabeled sample $x_{j}$, the prediction confidence $c_{j}=\max_{k}p_{k}^{\left(j\right)}$ is compared against a dynamic threshold $\tau_{\mathrm{dyn}}$ computed via Equation (9). Samples with $c_{j}<\tau_{\mathrm{dyn}}$ are rejected. **Stage 2 (KNN Graph):** Fused features $f_{j}=\mathrm{GAP}\left(Z_{\mathrm{fused}}^{\left(j\right)}\right)$ are used to construct a *k*-nearest neighbor graph based on cosine similarity, yielding normalized adjacency $\tilde{A}$. **Stage 3 (Consistency Check):** Neighborhood-aggregated predictions ${\bar{p}}_{i}=\sum_{j\in N_{k}\left(i\right)}{\tilde{A}}_{ij}p_{j}$ determine acceptance; pseudolabels enter P only when $\arg\max_{k}{\bar{p}}_{ik}={\hat{y}}_{i}$, ensuring that the local consensus matches the point prediction.

To leverage unlabeled data while avoiding error propagation from noisy pseudo-labels, we propose a graph-regularized acquisition strategy that combines confidence filtering with feature-space consistency verification. The pipeline is illustrated in Figure 4 and consists of two key components.

For each unlabeled sample $x_{j}\in D_{u}$, we compute the prediction confidence as $c_{j}=\max_{k}p_{k}^{\left(j\right)}$. A fixed threshold ignores that different categories have varying learning difficulties, which makes difficult categories less likely to be selected when filtering pseudolabeled samples. Therefore, we employ a dynamic threshold that adapts to the confidence distribution:

(9) $$\tau_{dyn}=\tau_{base}+\alpha \cdot {\left(\mathrm{Median}\left(\left\{c_{j}\right\}\right)-\tau_{base}\right)}^{+}$$

where $\tau_{base}$ is the base threshold, α is the blending coefficient, and ${\left(\cdot \right)}^{+}$ denotes the positive part function. This adaptive threshold adjusts to the model’s overall confidence level, ensuring balanced selection across categories with different learning difficulties. Samples exceeding this threshold form the candidate set C.

To verify pseudolabel quality through spatial–spectral consistency, we construct a *k*-nearest neighbor graph in the learned feature space. For each candidate sample, we extract the globally averaged fused feature $f_{j}=\mathrm{GAP}\left(Z_{fused}^{\left(j\right)}\right)\in R^{2D}$ and compute the cosine similarity to identify neighbors.

The neighborhood-aggregated prediction provides a smoothed estimate:

(10) $${\bar{p}}_{i}=\sum_{j\in N_{k}\left(i\right)}{\tilde{A}}_{ij}p_{j}$$

where $N_{k}\left(i\right)$ denotes the *k* nearest neighbors and ${\tilde{A}}_{ij}$ is the row-normalized adjacency weight. A pseudolabel is accepted only if the aggregated neighborhood prediction agrees with the sample’s own prediction:

(11) $$P=\{\left(x_{i},{\hat{y}}_{i}\right):c_{i}\geq \tau_{dyn}\;\mathrm{and}\;\arg\max_{k}{\bar{p}}_{ik}={\hat{y}}_{i}\}.$$

This graph consistency check effectively filters out samples near class boundaries or in ambiguous regions, ensuring that only high-quality pseudolabels contribute to model training.

#### 3.3.3. Stage 3: Semi-Supervised Refinement

After pseudolabel generation, HMGF-Net is fine-tuned on the augmented dataset $D_{aug}=D_{l}\cup P$. To account for potential noise in pseudolabels, we apply confidence-based sample reweighting, where labeled samples receive a weight of 1.0 and pseudolabeled samples receive a weight equal to their confidence score $c_{i}$. Training proceeds with a reduced learning rate $\eta_{ssl}=\gamma \cdot \eta_{base}$ (where γ∈[0.03,0.05]) to prevent catastrophic forgetting of the knowledge learned in Stage 1.

## 4. Results

This section presents a comprehensive evaluation of the proposed HMGF-Net with MS-PLR across three benchmark datasets: Houston2013, Augsburg, and Trento. We first describe the evaluation protocol, then present quantitative results with detailed comparisons against state-of-the-art methods, followed by parameter sensitivity studies and visual assessment.

### 4.1. Evaluation Protocol

The baseline methods span diverse state-of-the-art paradigms published between 2021–2024, including CNN-based approaches (Res-CP [30], CCR-Net [31], SepDGConv [32]), few-shot and semi-supervised methods (DCFSL [33], S3Net [34]), dual-modality fusion architectures (DSCA-Net [35], Fusion_HCT [36]), and transformer-based multimodal fusion (MFT [37]). Notably, MFT [37] (2023) represents the current state-of-the-art in multimodal remote sensing transformers, while DSCA-Net [35] (2024) is among the most recent dual-stream adaptive networks. This selection ensures a comprehensive comparison, with seven of eight baselines published in 2022 or later.

All models operated under identical training conditions with the same hyperspectral and LiDAR input modalities. The proposed HMGF-Net employed its dual-branch encoder, Mamba-based fusion module, and MS-PLR training strategy as described in Section 3.

Table 3 reports the hyperparameter configurations for MS-PLR. The base threshold $\tau_{\mathrm{base}}$ and KNN neighborhood size *k* were tuned per dataset via grid search, while the blending coefficient α=0.3 generalized well across all datasets without per-dataset adjustment. Houston2013 requires a higher $\tau_{\mathrm{base}}$ (0.60) due to its fifteen-class complexity and finer inter-class boundaries, whereas Trento and Augsburg benefit from larger *k* (20) owing to their spatially homogeneous agricultural and urban parcels. The KNN graph is reconstructed at the start of each SSL round using updated fused features, ensuring that neighborhood relationships reflect the current model state.

### 4.2. Quantitative Results

#### 4.2.1. Results on Houston2013

Table 4 presents the complete class-wise and overall results on Houston2013. The proposed HMGF-Net achieves the highest OA (92.30%), AA (93.43%), and Kappa (91.68), outperforming all comparison models. Notably, HMGF-Net demonstrates superior performance on challenging urban classes such as Commercial (94.72%), Residential (98.04%), and Road (91.51%), which exhibit high intra-class variability. The integration of Mamba-based long-range modeling with graph fusion provides stronger context propagation, while MS-PLR reduces pseudolabel noise near class boundaries.

#### 4.2.2. Results on Augsburg

Table 5 reports results on the Augsburg dataset, which contains large-scale and highly heterogeneous urban–vegetation mixtures. HMGF-Net achieves an OA of 88.61%, AA of 78.46%, and Kappa of 83.74, outperforming competing approaches. The Augsburg dataset poses significant challenges due to the dominance of the Residential-Area and Low-Plants classes, which together comprise over 70% of the scene. HMGF-Net significantly improves on Low-Plants (95.36%) and maintains competitive performance across minority classes. The graph-based fusion mechanism effectively incorporates elevation discontinuities and spectral relationships, ensuring reliable pseudolabel propagation.

#### 4.2.3. Results on Trento

Table 6 shows results on the Trento dataset. The proposed method achieves the highest OA (99.39%), AA (98.68%), and Kappa (99.18). Trento consists primarily of agricultural and semi-structured terrain where hyperspectral-LiDAR fusion plays a critical role in distinguishing vegetation types. HMGF-Net produces near-perfect accuracy for classes such as Apple Trees (99.70%), Woods (100.00%), Vineyard (99.98%), and Roads (96.97%). The Mamba state-space module effectively captures long-range spectral patterns, while the KNN graph consistency verification incorporates elevation and spatial continuity across agricultural parcels.

### 4.3. Comparative Analysis

Across all three datasets, HMGF-Net consistently surpasses existing models in OA, AA, and Kappa. Table 7 summarizes the performance comparison. The improvements arise from four key architectural and methodological strengths:

1. Hybrid Encoder Design: The 3D–2D CNN with residual connections for HSI and dense connections for LiDAR effectively captures modality-specific characteristics while maintaining computational efficiency.
2. Efficient Sequence Modeling: The Mamba block models long-range dependencies with O(L) complexity, offering advantages over standard CNNs (limited receptive field) and transformers ($O(L^{2})$ complexity).
3. Graph-Regularized Fusion: The KNN graph consistency verification aligns predictions semantically in the learned feature space, improving robustness against noisy pseudolabels.
4. Progressive Refinement: The MS-PLR strategy progressively expands the training set with validated pseudolabels, enabling effective utilization of unlabeled data under extreme label scarcity.

**Table 7:** Summary of HMGF-Net classification performance (%) across all datasets.

Dataset	OA	AA	Kappa
Houston2013	92.30	93.43	91.68
Augsburg	88.61	78.46	83.74
Trento	99.39	98.68	99.18

### 4.4. Parameter Sensitivity Analysis

To investigate the robustness of HMGF-Net to hyperparameter selection, we conducted systematic sensitivity analysis on three critical parameters: KNN neighborhood size *k*, LiDAR fusion weight $\omega_{l}$, and learning rate η. For all experiments, the batch size and patch size were fixed at 32 and 11×11, respectively, with label smoothing ϵ=0.1.

#### 4.4.1. Impact of KNN Neighborhood Size

**Figure 5:**
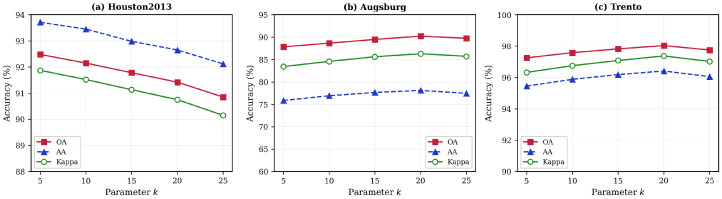
Impact of KNN neighborhood size on classification performance: (**a**) Houston2013; (**b**) Augsburg; (**c**) Trento.

The KNN neighborhood size *k* determines the scope of spatial–spectral consistency verification in the graph-regularized pseudolabel acquisition module. As illustrated in Figure 5, we evaluated classification performance with *k* values ranging from 5 to 25.

For Houston2013, optimal performance is achieved at k=5 (OA: 92.30%). This is attributed to its high spatial resolution (2.5 m), where neighboring pixels are more likely to belong to the same category within a compact neighborhood. In contrast, Augsburg and Trento achieve optimal results at k=20, reflecting their larger homogeneous regions that benefit from broader neighborhood context. These results demonstrate that optimal *k* is dataset-dependent and should be tuned based on spatial characteristics.

#### 4.4.2. Impact of LiDAR Fusion Weight

**Figure 6:**
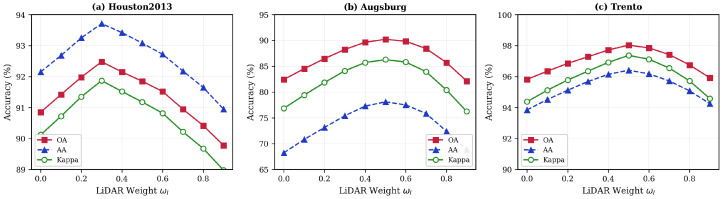
Impact of LiDAR fusion weight on classification performance: (**a**) Houston2013; (**b**) Augsburg; (**c**) Trento.

The fusion weight $\omega_{l}$ controls the relative contribution of LiDAR features, with HSI weight satisfying $\omega_{h}=1-\omega_{l}$. Figure 6 shows classification performance as $\omega_{l}$ varies from 0 to 0.9.

For Houston2013, optimal performance occurs at $\omega_{l}=0.3$, indicating that spectral information dominates for distinguishing diverse urban categories. For Augsburg and Trento, balanced fusion ($\omega_{l}=0.5$) yields the best results, as elevation information provides crucial discriminative features for separating vegetation types and distinguishing buildings. Performance degradation at extreme weights ($\omega_{l}=0$ or 0.9) confirms the importance of multimodal fusion.

#### 4.4.3. Impact of Learning Rate

**Figure 7:**
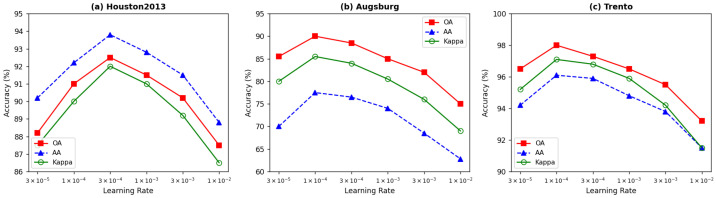
Impact of learning rate on classification performance: (**a**) Houston2013; (**b**) Augsburg; (**c**) Trento.

Figure 7 evaluates six learning rates: $\left\{3\times 10^{-5},10^{-4},3\times 10^{-4},10^{-3},3\times 10^{-3},10^{-2}\right\}$. Houston2013 achieves optimal performance at $3\times 10^{-4}$, while Augsburg and Trento perform best at $10^{-4}$.

The larger optimal learning rate for Houston2013 may be attributed to its complex fifteen-class feature space requiring more aggressive parameter updates. Excessively large learning rates ($10^{-2}$) cause significant performance degradation across all datasets, particularly for Augsburg (OA drops to 75.82%) due to its severe class imbalance. We recommend learning rates in the range $\left[10^{-4},3\times 10^{-4}\right]$ for similar HSI-LiDAR classification tasks.

### 4.5. Visual Assessment

**Figure 8:**
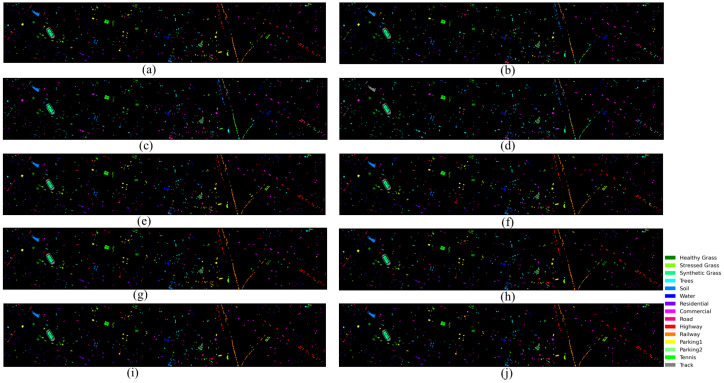
Classification maps for Houston2013: (**a**) ground truth; (**b**) Res-CP; (**c**) DCFSL; (**d**) S3Net; (**e**) DSCA-Net; (**f**) CCR-Net; (**g**) SepDGConv; (**h**) Fusion_HCT; (**i**) MFT; (**j**) proposed HMGF-Net (92.30%).

**Figure 9:**
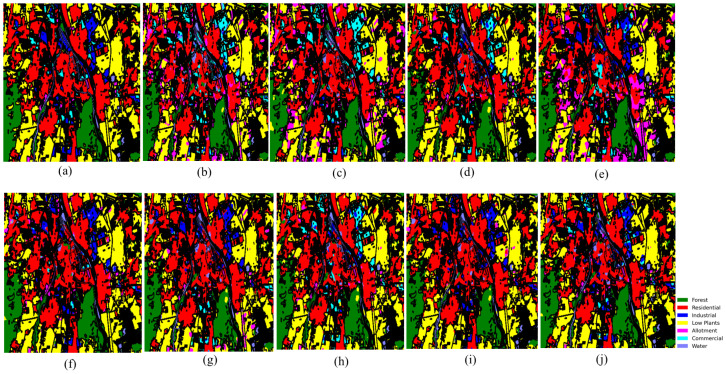
Classification maps for Augsburg: (**a**) ground truth; (**b**) Res-CP; (**c**) DCFSL; (**d**) S3Net; (**e**) DSCA-Net; (**f**) CCR-Net; (**g**) SepDGConv; (**h**) Fusion_HCT; (**i**) MFT; (**j**) proposed HMGF-Net (88.61%).

**Figure 10:**
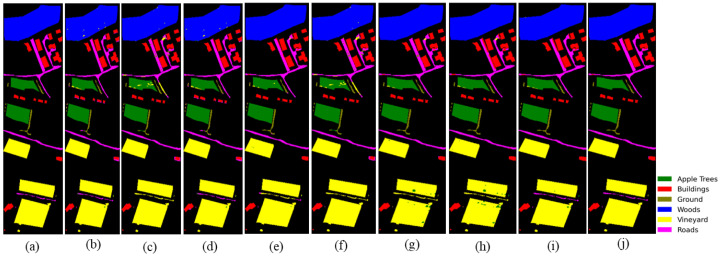
Classification maps for Trento: (**a**) ground truth; (**b**) Res-CP; (**c**) DCFSL; (**d**) S3Net; (**e**) DSCA-Net; (**f**) CCR-Net; (**g**) SepDGConv; (**h**) Fusion_HCT; (**i**) MFT; (**j**) proposed HMGF-Net (99.39%).

Figure 8, Figure 9 and Figure 10 present visual classification maps for all three datasets. Compared to baseline methods, HMGF-Net produces smoother regions, cleaner class boundaries, and fewer isolated misclassifications.

On Houston2013, HMGF-Net exhibits improved discrimination along road networks and parking lot boundaries, where spectral confusion is prevalent. The Augsburg results show enhanced separation between residential and commercial areas, benefiting from the elevation-aware fusion mechanism. On Trento, the agricultural parcel boundaries are sharply delineated, demonstrating effective utilization of both spectral signatures and terrain structure.

### 4.6. Summary

The experimental results across the Houston2013, Augsburg, and Trento datasets confirm that HMGF-Net with MS-PLR offers substantial advantages in multimodal semi-supervised learning. Through its combination of spectral–spatial encoding, Mamba-based sequence modeling, graph-regularized fusion, and progressive pseudolabel refinement, the proposed framework delivers robust performance under limited annotation, consistently surpassing state-of-the-art methods across all datasets and evaluation metrics.

## 5. Discussion

### 5.1. Ablation Study

**Figure 11:**
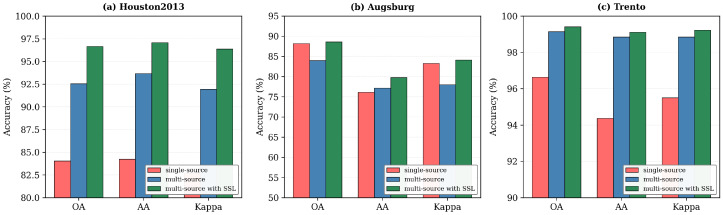
Ablation study on the effectiveness of semi-supervised learning: (**a**) Houston2013; (**b**) Augsburg; (**c**) Trento.

**Figure 12:**
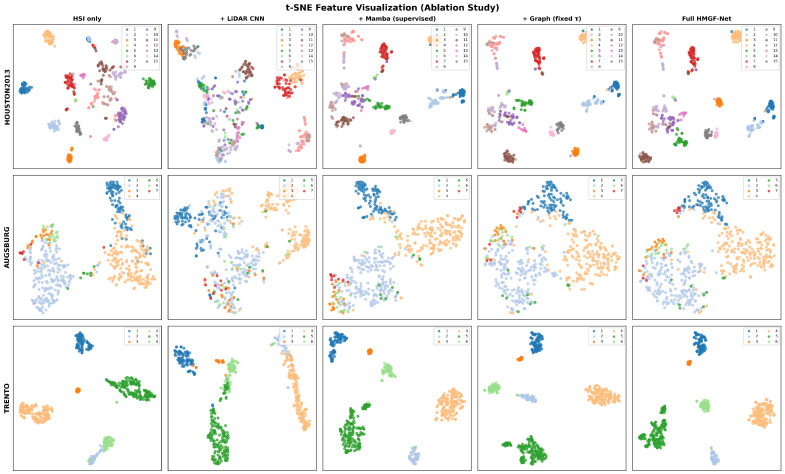
t-SNE visualization of learned feature representations across different ablation configurations. Each row corresponds to a dataset (Houston2013, Augsburg, Trento) and each column represents an ablation variant (HSI only, + LiDAR CNN, + Mamba, + Graph, Full HMGF-Net). Different colors indicate different land cover categories.

To comprehensively evaluate the contribution of each component in our proposed HMGF-Net framework, we conducted extensive ablation experiments on all three benchmark datasets. The ablation study is organized into four parts: (1) module-wise quantitative analysis, presented in Table 8; (2) semi-supervised learning effectiveness, illustrated in Figure 11; (3) feature representation visualization using t-SNE, shown in Figure 12; and (4) computational complexity comparison, reported in Table 9.

#### 5.1.1. Module-Wise Ablation Analysis

To quantify the contribution of each component in our proposed framework, we conducted a comprehensive module-wise ablation study. The results are presented in Table 8. Starting from the HSI-only baseline, we progressively integrated each module and evaluate the resulting classification performance across all three datasets. The HSI-only baseline, which employs only the spectral–spatial encoder without any auxiliary information, achieves OA of 96.64%, 84.04%, and 88.18% on the Trento, Houston2013, and Augsburg datasets, respectively. In contrast, using LiDAR data alone yields significantly lower performance with OA of 90.24%, 61.45%, and 58.61%, confirming that spectral information from HSI provides more discriminative features than elevation information alone for land cover classification tasks.

Introduction of the Mamba-based fusion module, which integrates both HSI and LiDAR inputs through state-space sequence modeling, substantially improves the classification performance, achieving OA of 99.14%, 92.54%, and 83.96% on the three datasets. This improvement of 2.50%, 8.50%, and −4.22% over HSI-only baseline demonstrates the effectiveness of our cross-modal fusion strategy in capturing long-range spatial dependencies and complementary information between modalities. The temporary performance drop on the Augsburg dataset can be attributed to the introduction of noisy elevation features in heterogeneous urban regions, which is subsequently addressed by the graph-based refinement. Adding the graph-based consistency verification with a fixed threshold further enhances the results to 99.38%, 95.31%, and 88.46% OA, highlighting the importance of pseudolabel quality control through spatial–spectral neighborhood consistency checking.

The performance pattern on Augsburg (88.18% → 83.96% → 88.61%) warrants detailed analysis. The temporary drop after introducing the Mamba fusion module is caused by LiDAR noise at 30 m resolution inducing modality-fusion mismatch, rather than by oversmoothing from sequence modeling. Three observations support this conclusion: (1) LiDAR-only classification achieves only 58.61% OA on Augsburg, the lowest across all datasets, confirming limited discriminative power at coarse resolution; (2) Augsburg’s urban classes exhibit spectral homogeneity but elevation heterogeneity, causing noisy LiDAR features to dilute discriminative spectral information when fused; and (3) if oversmoothing were responsible, degradation would occur across all datasets, yet Houston2013 shows +8.50% improvement and Trento achieves 99.38% OA. The graph-consistency verification addresses this by rejecting pseudolabels where neighborhood predictions disagree, recovering OA from 83.96% to 88.46% (+4.50%).

Finally, the complete HMGF-Net with the proposed MS-PLR strategy achieves the best performance across all datasets: 99.41% OA on Trento (+2.77% over baseline), 96.65% OA on Houston2013 (+12.61% over baseline), and 88.61% OA on Augsburg (+0.43% over baseline). The most significant improvement is observed on the Houston2013 dataset, where the complex urban environment with fifteen diverse land cover categories benefits substantially from our multimodal fusion and semi-supervised learning approach. These ablation results validate that each proposed component contributes positively to the final classification performance and that their synergistic combination yields optimal results across diverse remote sensing scenarios.

#### 5.1.2. Effectiveness of Semi-Supervised Learning

To further validate the effectiveness of our proposed semi-supervised learning strategy, we compared three representative configurations, as illustrated in Figure 11: single-source classification using only HSI data, multi-source fusion combining HSI and LiDAR through the Mamba-based module under fully supervised settings, and multi-source with SSL, representing the complete HMGF-Net framework with graph-regularized pseudolabel acquisition. The visualization clearly demonstrates the progressive performance improvement achieved by each enhancement. On the Houston2013 dataset, the multi-source configuration achieves OA of 92.54%, AA of 93.66%, and Kappa of 91.94%, representing substantial improvements of 8.50%, 9.42%, and 9.22% over the single-source baseline (OA = 84.04%, AA = 84.24%, Kappa = 82.72%). Incorporation of our semi-supervised learning strategy with MS-PLR further boosts the performance to OA of 96.65%, AA of 97.08%, and Kappa of 96.37%, demonstrating the significant benefit of leveraging abundant unlabeled samples through propagation of high-confidence pseudolabels.

Similar performance gains are observed for the Trento dataset, where the full HMGF-Net achieves 99.41% OA compared to 96.64% for single-source and 99.14% for multi-source without SSL. The consistent improvement across all three metrics confirms that the semi-supervised refinement effectively enhances the model’s generalization capability by exploiting the rich spatial structure in unlabeled data. For the Augsburg dataset, which presents more challenging scenarios with severe class imbalance and complex urban landscapes, multi-source fusion initially shows slightly lower OA (83.96%) than single-source (88.18%) due to the introduction of noisy elevation features in heterogeneous regions. However, the semi-supervised refinement stage effectively addresses this limitation by selectively incorporating reliable pseudolabels verified through graph-based spatial consistency, ultimately achieving 88.61% OA with notably improved AA (79.75% vs. 76.10%) and Kappa coefficient (84.13% vs. 83.27%). These experimental results confirm that our proposed MS-PLR strategy successfully exploits the complementary information from both modalities while leveraging unlabeled samples to enhance classification accuracy across diverse remote sensing scenarios.

#### 5.1.3. Feature Representation Visualization

To provide intuitive insights into the discriminative capability of learned feature representations, we employ t-distributed Stochastic Neighbor Embedding (t-SNE) to visualize the high-dimensional features extracted by different ablation configurations, as shown in Figure 12. Each row corresponds to a specific dataset (Houston2013, Augsburg, and Trento, from top to bottom), while each column represents a progressive ablation variant from left to right: HSI only, + LiDAR CNN, + Mamba (supervised), + Graph (fixed τ), and Full HMGF-Net.

For the Houston2013 dataset with fifteen land cover categories, the HSI-only baseline exhibits considerable overlap between semantically similar classes, particularly among different vegetation types and urban structures. The introduction of LiDAR features initially increases inter-class confusion due to the limited discriminative power of elevation information alone. However, the Mamba-based fusion module effectively integrates complementary cross-modal information, resulting in more compact intra-class clusters and clearer inter-class boundaries. The subsequent graph-based refinement and full HMGF-Net configuration progressively improve the cluster separation, with the final visualization showing well-defined, tightly grouped clusters for all fifteen categories with minimal overlap.

Similar progressive improvements are observed for the Augsburg dataset with seven classes. The initial HSI-only representation shows significant mixing between classes 1–2 (different forest types) and classes 3–4 (residential and industrial areas). The Mamba fusion and graph-based refinement stages gradually disentangle these confusing categories, with the full HMGF-Net producing the most separable feature space where each class forms a distinct and compact cluster. For the Trento dataset with six classes, the relatively simpler classification task results in well-separated clusters even with the baseline configuration. Nevertheless, the progressive integration of modules further enhances the compactness of intra-class distributions and increases the margins between different categories. The full HMGF-Net achieves the most discriminative feature representation with clearly defined boundaries and minimal intra-class variance, which directly translates to the superior classification accuracy of 99.41% OA reported in Table 8. These visualizations provide compelling qualitative evidence that each proposed component contributes to learning more discriminative and well-structured feature representations for HSI-LiDAR classification.

#### 5.1.4. Computational Complexity Analysis

Table 9 compares the computational cost of all methods on Houston2013 in terms of trainable parameters, FLOPs, training time, and inference time. HMGF-Net contains only 227.63 K parameters, a reduction of 93.9% relative to DSCA-Net (3737.71 K) and 75.8% relative to MFT (940.79 K). The parameter efficiency arises from replacing quadratic self-attention ($O(L^{2})$) with linear state-space recurrence (O(L)) in the Mamba block. Training requires 688.64 s owing to the iterative pseudolabel refinement stages; however, inference completes in 2.07 s for the full test set, comparable to lightweight baselines such as DCFSL (2.04 s) and Fusion_HCT (2.35 s). Despite this modest computational footprint, HMGF-Net attains the highest OA (92.30%), outperforming DSCA-Net by +1.50% while using only 6.1% of its parameters, demonstrating a favorable accuracy–efficiency tradeoff that is suitable for operational deployment.

Compared to the transformer-based MFT, HMGF-Net reduces parameters by 75.8% (227.63 K vs. 940.79 K) while achieving +6.36% higher OA (92.30% vs. 85.94%), confirming the efficiency advantage of linear-complexity state-space modeling over quadratic self-attention.

### 5.2. Cross-Dataset Generalization Analysis

To assess the portability of HMGF-Net across heterogeneous remote sensing scenarios, we examined performance variations for semantically equivalent classes appearing in multiple datasets.

The Water class exhibits the largest cross-dataset variation, with 100.00% on Houston2013 versus 66.12% on Augsburg. Three factors account for this discrepancy:

1. Spatial Resolution: Houston2013 (2.5 m) resolves water bodies as spectrally pure pixels, whereas Augsburg (30 m) produces mixed pixels containing water, vegetation, and built-up materials along riverbanks and canals. Mixed pixels exhibit ambiguous spectral signatures that reduce classifier confidence.
2. Spectral Range: Augsburg spans 0.4–2.5 µm, including Short-Wave Infra-Red (SWIR) bands where water absorption is strong but variable depending on turbidity, depth, and dissolved constituents. Houston2013 covers only 0.38–1.05 µm, where water reflectance is more stable and distinctive.
3. Water Body Heterogeneity: Houston2013 contains relatively homogeneous urban water features (retention ponds, swimming pools), while Augsburg includes rivers, industrial waterways, and agricultural irrigation channels with diverse spectral characteristics.

Despite these challenges, HMGF-Net achieves the highest accuracy for the Water class on Augsburg among all compared methods (66.12% versus 65.99% for DSCA-Net and 41.78% for MFT), demonstrating robust generalization even under unfavorable imaging conditions. The consistent improvements across all three datasets, each with distinct sensors, resolutions, and land-cover distributions, confirm that the proposed architecture generalizes effectively without dataset-specific tuning. Future work may incorporate resolution-adaptive modules or domain adaptation techniques to further enhance cross-sensor transferability.

## 6. Conclusions

This work introduces HMGF-Net, a unified multimodal framework designed to address the challenges of semi-supervised hyperspectral–LiDAR classification under extremely limited labeled data. By combining a 3D–2D spectral–spatial encoder for hyperspectral imagery, a multi-scale CNN for LiDAR elevation modeling, and an efficient Mamba selective state-space module for long-range feature refinement, the network captures both local spectral–spatial structure and global contextual dependencies. A graph-based fusion mechanism further enhances cross-modal alignment by modeling relational consistency across spectral, spatial, and elevation domains. To ensure training stability in low-label scenarios, the proposed Multi-Stage Pseudo-Label Refinement (MS-PLR) framework progressively mitigates label noise through confidence filtering, spatial–spectral smoothing, and graph-consistency propagation.

Extensive experiments on the Houston2013, Augsburg, and Trento datasets demonstrate that HMGF-Net consistently outperforms state-of-the-art hyperspectral, multimodal, and semi-supervised learning approaches. The model achieves superior overall accuracy, average accuracy, and Kappa values across all datasets, with notable improvements in structurally complex or spectrally ambiguous classes. The results confirm that integrating selective state-space modeling with graph-guided fusion and progressive pseudolabel refinement offers a robust and efficient solution for multimodal classification under restricted supervision.

Future research may extend the framework toward large-scale scene understanding, real-time inference, and multimodal transformer–state-space hybrids. Moreover, the integration of physics-informed priors, domain generalization mechanisms, or additional modalities such as SAR and multispectral data may further broaden the applicability of the proposed approach in operational remote sensing environments.

## Figures and Tables

**Table 1:** Dataset description.

Dataset	Houston2013 [27]		Trento [28]		Augsburg [29]	
Location	Houston, Texas, USA		Trento, Italy		Augsburg, Germany	
Sensor Type	HSI	LiDAR	HSI	LiDAR	HSI	LiDAR
Image Size	349 × 1905	349 × 1905	600 × 166	600 × 166	332 × 485	332 × 485
Spatial Resolution	2.5 m	2.5 m	1 m	1 m	30 m	30 m
Number of Bands	144	1	63	1	180	1
Wavelength Range	0.38–1.05 m	/	0.42–0.99 m	/	0.4–2.5 m	/
Sensor Name	CASI-1500	/	AISA Eagle	Optech ALTM 3100EA	HySpex	DLR-3 K

**Table 2:** Summary of the notation used in this paper.

Symbol	Description	Symbol	Description
*Input and Output*			
$X_{H}$	HSI cube (H×W×C)	$X_{L}$	LiDAR DSM (H×W×1)
*K*	Number of land-cover classes	*P*	Patch size
$D_{l}$	Labeled training set	$D_{u}$	Unlabeled sample pool
*Feature Extraction*			
$F_{H}^{\left(l\right)}$	HSI feature at layer *l*	$F_{L}^{\left(l\right)}$	LiDAR feature at layer *l*
$Z_{H}$	Encoded HSI features	$Z_{L}$	Encoded LiDAR features
*D*	Feature dimension (64)	H(·)	Layer mapping function
*Mamba Fusion Module*			
$Z_{cat}$	Concatenated features	S	Sequence representation
*L*	Sequence length ($P^{2}$)	*N*	Feature channels (2D)
$h_{t}$	Latent state at position *t*	$N_{s}$	State dimension
$\bar{A}$	Discretized state matrix	${\bar{B}}_{t},C_{t}$	Input-dependent projections
$Z_{fused}$	Fused output features	$\sigma_{g}$	SiLU activation
*Pseudo-Label Acquisition*			
$c_{j}$	Prediction confidence	$\tau_{base}$	Base threshold
$\tau_{dyn}$	Dynamic threshold	α	Blending coefficient
$N_{k}(i)$	*k*-nearest neighbors of *i*	${\tilde{A}}_{ij}$	Normalized adjacency
${\bar{p}}_{i}$	Aggregated prediction	P	Validated pseudolabel set
*Training Parameters*			
$L_{sup}$	Supervised loss	ϵ	Label smoothing (0.1)
η	Base learning rate	γ	LR reduction factor
$E_{sup}$	Supervised epochs	$E_{ssl}$	SSL refinement epochs

**Table 3:** Hyperparameter settings for MS-PLR across datasets. Values were selected via grid search on a held-out validation set (10% of training samples) *.

Hyperparameter	Houston2013	Trento	Augsburg
Base threshold $\tau_{\mathrm{base}}$	0.60	0.50	0.55
Blending coefficient α	0.3	0.3	0.3
KNN neighborhood *k*	5	20	20
Graph update frequency	Every SSL round	Every SSL round	Every SSL round
Supervised epochs $E_{\sup}$	100	80	100
SSL refinement epochs $E_{\mathrm{ssl}}$	15	20	15

* Search ranges: $\tau_{\mathrm{base}}\in \left[0.40,0.70\right]$; α∈[0.1,0.5]; k∈[5,25].

**Table 4:** Comparison of classification accuracy (%) of different methods on the Houston2013 dataset. The competing methods include Res-CP [30], DCFSL [33], S3Net [34], DSCA-Net [35], CCR-Net [31], SepDGConv [32], Fusion_HCT [36], and MFT [37]. The best results are highlighted in **bold**.

No.	Class (Train/Test)	Res-CP	DCFSL	S3Net	DSCA-Net	CCR-Net	SepDGConv	Fusion_HCT	MFT	Proposed
1	Healthy grass (10/1241)	98.51	96.21	94.04	93.31	83.40	87.37	82.35	93.39	84.03
2	Stressed grass (10/1244)	87.96	96.62	91.48	92.77	83.68	86.60	99.52	81.99	**100.00**
3	Synthetic grass (10/687)	**100.00**	98.98	**100.00**	98.98	98.25	98.85	97.09	99.56	**100.00**
4	Trees (10/1234)	98.07	96.52	95.79	88.74	87.60	75.72	96.27	92.22	93.28
5	Soil (10/1232)	99.47	99.19	95.13	**100.00**	95.29	97.18	**100.00**	99.35	98.89
6	Water (10/315)	**100.00**	82.86	98.41	89.84	86.98	92.62	**100.00**	98.09	**100.00**
7	Residential (10/1258)	93.39	87.52	82.19	93.64	70.11	74.68	76.39	85.93	**98.04**
8	Commercial (10/1234)	67.76	65.88	69.04	75.12	58.83	59.24	75.93	73.99	**94.72**
9	Road (10/1242)	87.41	80.27	74.48	**91.38**	50.56	56.31	87.20	69.40	91.51
10	Highway (10/1217)	73.79	**81.68**	76.99	78.80	57.76	56.89	81.92	65.16	74.59
11	Railway (10/1225)	91.16	80.73	**94.37**	90.12	54.12	78.29	90.78	85.79	93.86
12	Parking lot 1 (10/1223)	71.74	**93.87**	75.31	89.29	73.02	81.59	59.52	92.80	79.39
13	Parking lot 2 (10/459)	87.84	98.26	94.77	**99.78**	69.72	89.98	86.27	79.74	93.21
14	Tennis court (10/418)	98.12	98.33	**100.00**	**100.00**	90.19	92.29	**100.00**	**100.00**	**100.00**
15	Running track (10/650)	92.07	99.23	**100.00**	98.00	76.15	98.03	**100.00**	97.54	**100.00**
	OA (%)	87.31	89.38	87.26	90.80	73.71	78.79	87.00	85.94	**92.30**
	AA (%)	89.82	90.41	89.47	91.99	75.71	81.71	88.88	87.66	**93.43**
	Kappa (×100)	86.28	88.52	86.24	90.06	71.66	77.11	85.95	84.80	**91.68**

**Table 5:** Comparison of classification accuracy (%) of different methods on the Augsburg dataset. The competing methods include Res-CP [30], DCFSL [33], S3Net [34], DSCA-Net [35], CCR-Net [31], SepDGConv [32], Fusion_HCT [36], and MFT [37]. The best results are highlighted in **bold**.

No.	Class (Train/Test)	Res-CP	DCFSL	S3Net	DSCA-Net	CCR-Net	SepDGConv	Fusion_HCT	MFT	Proposed
1	Forest (10/13,497)	90.50	91.74	**98.53**	93.91	89.61	83.08	93.37	94.66	97.82
2	Residential-Area (10/30,319)	**98.66**	70.27	86.59	87.62	45.08	71.19	87.84	92.82	86.45
3	Industrial-Area (10/3841)	**77.17**	52.90	50.19	63.08	46.99	45.44	24.24	68.68	61.28
4	Low-Plants (10/26,847)	99.58	67.50	56.79	81.81	47.10	47.83	67.73	90.17	**95.36**
5	Allotment (10/565)	20.89	**87.96**	73.98	69.38	86.19	72.52	79.82	33.63	79.65
6	Commercial-Area (10/1635)	17.21	42.87	55.35	47.65	37.19	32.21	**71.44**	18.84	62.54
7	Water (10/1520)	39.54	62.50	65.65	65.99	52.70	42.42	59.74	41.78	**66.12**
	OA (%)	83.36	71.57	75.48	84.12	53.83	62.59	77.82	88.07	**88.61**
	AA (%)	63.37	67.96	69.59	72.78	57.84	56.39	69.17	62.93	**78.46**
	Kappa (×100)	77.38	62.99	67.05	77.94	42.56	50.33	69.37	82.93	**83.74**

**Table 6:** Comparison of classification accuracy (%) of different methods on the Trento dataset. The competing methods include Res-CP [30], DCFSL [33], S3Net [34], DSCA-Net [35], CCR-Net [31], SepDGConv [32], Fusion_HCT [36], and MFT [37]. The best results are highlighted in **bold**.

No.	Class (Train/Test)	Res-CP	DCFSL	S3Net	DSCA-Net	CCR-Net	SepDGConv	Fusion_HCT	MFT	Proposed
1	Apple trees (10/4024)	98.46	99.58	89.46	99.85	98.31	88.57	98.73	96.94	**99.70**
2	Building (10/2893)	90.23	89.70	73.56	78.64	95.06	98.97	90.94	**99.89**	97.77
3	Ground (10/469)	98.71	**99.79**	97.87	98.08	81.88	97.28	97.65	83.37	97.63
4	Woods (10/9113)	99.98	98.95	81.75	**100.00**	99.62	99.25	**100.00**	99.84	**100.00**
5	Vineyard (10/10,491)	99.28	98.73	72.90	**100.00**	88.79	91.47	92.76	98.83	99.98
6	Roads (10/3164)	64.89	82.33	81.48	82.02	88.15	92.31	84.54	83.57	**96.97**
	OA (%)	93.63	96.34	79.14	96.01	94.38	94.34	94.78	97.14	**99.39**
	AA (%)	91.92	94.85	82.84	93.10	92.13	94.64	94.11	93.74	**98.68**
	Kappa (×100)	91.50	95.12	72.27	94.68	92.21	92.48	93.10	96.19	**99.18**

**Table 8:** Module-wise ablation of HMGF-Net on three benchmark datasets. Ticks (✓) indicate enabled modules; crosses (✗) indicate disabled modules. The best results are shown in **bold**.

Variant	HSI	LiDAR	Mamba	Graph	MS–PLR	Trento			Houston2013			Augsburg		
						OA	AA	κ	OA	AA	κ	OA	AA	κ
HSI only (baseline)	✓	✗	✗	✗	✗	96.64	94.37	95.50	84.04	84.24	82.72	88.18	76.10	83.77
LiDAR CNN only	✗	✓	✗	✗	✗	90.24	90.64	87.26	61.45	60.89	58.43	58.61	58.97	48.66
+ Mamba block	✓	✓	✓	✗	✗	99.38	98.95	99.17	92.54	93.66	91.94	83.96	77.13	79.40
+ Graph fusion (fixed τ)	✓	✓	✓	✓	✗	99.38	**99.38**	99.19	95.31	96.04	94.93	88.46	79.24	83.91
Full HMGF-Net (MS–PLR)	✓	✓	✓	✓	✓	**99.41**	99.11	**99.22**	**95.97**	**96.34**	**95.60**	**88.61**	**79.75**	**84.13**

**Table 9:** Computational complexity comparison on the Houston2013 dataset. Params: trainable parameters (K); FLOPs: floating-point operations (M); Mem: peak GPU memory (GB); Train: total training time (s); Test: inference time for entire test set (s). Hardware: NVIDIA RTX 4090 (24 GB), 64 GB RAM (Santa Clara, CA, USA), batch size 32, patch size 11×11. Best results in **bold**.

Method	Year	Params (K)	FLOPs (M)	Mem (GB)	Train (s)	Test (s)	OA (%)
CCR-Net [31]	2021	70.08	0.14	1.2	61.12	0.06	73.71
Res-CP [30]	2022	180.02	2.23	1.8	115.77	1.56	87.31
S3Net [34]	2022	229.10	46.87	2.4	323.86	4.27	87.26
DCFSL [33]	2022	284.14	28.75	2.6	563.14	2.04	89.38
SepDGConv [32]	2022	312.45	18.64	2.2	198.42	1.82	78.79
Fusion_HCT [36]	2022	425.38	32.17	3.1	245.63	2.35	87.00
MFT [37]	2023	940.79	6.32	4.6	163.70	1.78	85.94
DSCA-Net [35]	2024	3737.71	322.52	8.4	472.35	5.81	90.80
**HMGF-Net (Ours)**	2025	227.63	52.48	2.8	688.64	2.07	**92.30**

Metrics reported on Houston2013 as representative benchmark; architecture and computational cost remain consistent across datasets, with <5% FLOPs variation due to input band differences (63–180 bands).

## Data Availability

The datasets used in this study are publicly available from their corresponding references. The code and trained models will be made available upon reasonable request to the corresponding author.
